# Elongin C Contributes to RNA Polymerase II Degradation by the Interferon Antagonist NSs of La Crosse Orthobunyavirus

**DOI:** 10.1128/JVI.02134-19

**Published:** 2020-03-17

**Authors:** Andreas Schoen, Simone Lau, Paul Verbruggen, Friedemann Weber

**Affiliations:** a Institute for Virology, FB10-Veterinary Medicine, Justus Liebig University, Giessen, Germany; b Institute for Virology, Philipps University Marburg, Marburg, Germany; c Institute of Virology, Medical Center University of Freiburg, Freiburg, Germany; St. Jude Children’s Research Hospital

**Keywords:** La Crosse virus, nonstructural protein NSs, RNA polymerase II degradation, Elongin C, La Crosse virus

## Abstract

Mosquito-borne La Crosse virus (LACV; genus *Orthobunyavirus*, family *Peribunyaviridae*, order *Bunyavirales*) causes up to 100 annual cases of severe meningoencephalitis in children and young adults in the United States. A major virulence factor of LACV is the nonstructural protein NSs, which inhibits host cell mRNA synthesis to prevent the induction of antiviral type I interferons (IFN-α/β). To achieve this host transcriptional shutoff, LACV NSs drives the proteasomal degradation of RPB1, the large subunit of mammalian RNA polymerase II. Here, we show that NSs acts in a surprisingly rapid manner, as RPB1 degradation was commencing already at 1 h postinfection. The RPB1 degradation was partially dependent on the cellular E3 ubiquitin ligase subunit Elongin C. Consequently, removal of Elongin C, but also of the subunits Elongin A or B by siRNA transfection partially rescued general RNAP II transcription and IFN-beta mRNA synthesis from the blockade by NSs. In line with these results, LACV NSs was found to trigger the redistribution of Elongin C out of nucleolar speckles, which, however, is an epiphenomenon rather than part of the NSs mechanism. Our study also shows that the molecular phenotype of LACV NSs is different from RNA polymerase II inhibitors like α-amanitin or Rift Valley fever virus NSs, indicating that LACV is unique in involving the Elongin complex to shut off host transcription and IFN response.

**IMPORTANCE** The mosquito-borne La Crosse virus (LACV; genus *Orthobunyavirus*, family *Peribunyaviridae*, order *Bunyavirales*) is prevalent in the United States and can cause severe childhood meningoencephalitis. Its main virulence factor, the nonstructural protein NSs, is a strong inhibitor of the antiviral type I interferon (IFN) system. NSs acts by imposing a global host mRNA synthesis shutoff, mediated by NSs-driven proteasomal degradation of the RPB1 subunit of RNA polymerase II. Here, we show that RPB1 degradation commences as early as 1 h postinfection, and identify the E3 ubiquitin ligase subunit Elongin C (and its binding partners Elongins A and B) as an NSs cofactor involved in RPB1 degradation and in suppression of global as well as IFN-related mRNA synthesis.

## Abstract (precis)

The mosquito-borne La Crosse virus (LACV; genus *Orthobunyavirus*, family *Peribunyaviridae*, order *Bunyavirales*) is prevalent in the United States and can cause severe childhood meningoencephalitis. Its main virulence factor, the nonstructural protein NSs, is a strong inhibitor of the antiviral type I interferon (IFN) system. NSs acts by imposing a global host mRNA synthesis shutoff, mediated by NSs-driven proteasomal degradation of the RPB1 subunit of RNA polymerase II. Here, we show that RPB1 degradation commences as early as 1 h postinfection, and identify the E3 ubiquitin ligase subunit Elongin C (and its binding partners Elongins A and B) as an NSs cofactor involved in RPB1 degradation and in suppression of global as well as IFN-related mRNA synthesis.

## INTRODUCTION

Members of the mosquito-borne Orthobunyaviruses, present all over the world, are getting increased awareness as a threat to human and animal health. The African type species Bunyamwera virus (BUNV), that *per se* causes a mild febrile illness in humans, has shown potential to convert into a hemorrhagic fever virus, called Ngari (1, 2). The asian-australian Akabane virus as well as the Schmallenberg virus (SBV) that has recently spread all over Western Europe, are causing stillbirths, abortions and congenital malformations in large numbers of ruminants (3, 4). Recurrent epidemics of debilitating fever due to Oropouche virus infections, raging for over 60 years, have affected more than half a million people in Latin America (5, 6). Also, Maguari-like viruses, associated with febrile illness, are infecting humans all over South America (7). Members of the Maputta serogroup are responsible for epidemics of an acute polyarthritis-like disease in Papua-New Guinea and Australia (8). La Crosse virus (LACV) is the causative agent of a severe meningoencephalitis that mostly (but not exclusively) affects children and young adults in the United States (9–12). Per year, up to 100 cases have required hospitalization or even intensive care, exceeding West Nile virus in numbers of pediatric neuroinvasive arboviral infection (13). A substantial proportion of patients are suffering from long-lasting neurological problems (11). Since most infections, especially in adults, are, however, mild or inapparent, the number of subclinical infections was estimated to be around 300,000 annually (14).

The group of orthobunyaviruses is taxonomically defined as a genus within the family *Peribunyaviridae*, order *Bunyavirales* (15). The pleomorphic virions are enveloped and have a diameter of approximately 100 nm. As is typical for bunyaviruses (16), their genome consists of three segments of negative-strand RNA that are named L (large; ca. 7,000 nucleotides [nt]), M (medium; ca. 4,500 nt), and S (small; ca 950 nt). The L segment encodes the RNA-dependent RNA polymerase (RdRP), the M segments encodes a polyprotein that is processed to the envelope glycoproteins Gn, NSm (nonstructural, M segment), and Gc, and the S segments encodes the nucleocapsid protein N and the nonstructural protein NSs. All genomic segments are encapsidated by N protein and contain noncoding regions at their 5′ and 3′ ends that have the potential to anneal to a so-called “panhandle structure” due to partial sequence complementarities. The panhandle sequences are bound by the L RdRP and constitute the promoter for viral mRNA transcription and genome replication (17).

The entire multiplication cycle of bunyaviruses takes place in the cytoplasm. After entering the host cell via clathrin-mediated endocytosis (18, 19) and subsequent low pH-driven membrane fusion, mRNAs are transcribed from the incoming genome RNA nucleocapsids by L RdRP (“primary transcription”). The transcription is primed by 12- to 18-nt 5′-capped oligonucleotides that had been cleaved from host mRNA by an endonuclease activity residing in the N terminus of L (20). After translation of the viral proteins, the viral genome RNA (vRNA) is replicated via a positive-sense, encapsidated full-length intermediate, the copy RNA (cRNA). The newly generated vRNA nucleocapsids can give rise to more mRNAs produced by secondary transcription or become packaged by peptidase-processed Gn/Gc on Golgi membranes and leave the cell via the exocytosis pathway (19, 21, 22).

For orthobunyaviruses, the S segment-encoded protein NSs is a major determinant of pathogenicity, acting as an antagonist of the antiviral type I interferon (IFN) response (19, 23). IFNs are cytokines that become produced upon virus detection by host cells and stimulate the expression of genes (ISGs) for proteins with antiviral activity (24, 25). In the case of LACV, infection is detected by the IFN system via the RIG-I/MAVS virus sensor axis (26–28). RIG-I, a cytoplasmic RNA helicase (29, 30), is capable of recognizing the panhandle RNA of bunyaviruses, even if packaged by nucleocapsids (31, 32). RIG-I-mediated panhandle detection activates the transcription factor IRF-3, leading to the production of IFN-β mRNA (33, 34). It is known that orthobunyavirus multiplication is affected by IFN (35–38), and several ISGs were shown to be involved in this antiviral activity (36, 39–42). To counteract the IFN/ISG induction, the orthobunyavirus NSs, however, massively and rapidly inhibits cellular mRNA transcription, leaving the host unable to appropriately respond to the infection (35, 43). We have previously shown that the NSs of the orthobunyaviruses BUNV and LACV directly interfere with mRNA synthesis by the cellular RNA polymerase II (RNAP II) (28, 44). While BUNV NSs is reducing the mRNA elongation-relevant phosphorylation of the C-terminal serine 2 residue (part of the 52 times repeated heptapeptide motif in the C-terminal domain [CTD]) (44), LACV NSs additionally degrades the large subunit (RPB1) of RNAP II (28). An RPB1-degradative activity was also described for the NSs of SBV (43, 45). Interestingly, the effect of LACV NSs on RPB1 has strong similarities with parts of the DNA damage response (DDR), not only in terms of RPB1 degradation but also by activation of other DDR markers (28).

The impact of orthobunyavirus NSs on IFN induction and its biological relevance are well established (19). Mechanistically, however, far less is known. Here, we further investigated the effect that NSs has on the cells and on RNAP II, and we identified Elongin C and the Elongin complex as a host factor involved in NSs action.

## RESULTS

### LACV NSs rapidly reduces RNAP IIo.

To become transcriptionally active, the 260-kDa large subunit RPB1 of RNAP II gets hyperphosphorylated at the 52 heptad repeat sequences that are situated at the CTD (46, 47). This results in a gain of molecular weight and hence in a band shift on immunoblots. The NSs proteins of both the orthobunyavirus type species BUNV and of LACV trigger the disappearance of the high-molecular-weight hyperphosphorylated RNAP II, termed IIo, and with some delay also of the lighter nonphosphorylated, transcriptionally inactive form (IIa) (28, 44). Moreover, the RPB1 CTD has two major phosphorylation sites within each of the 52 heptad repeats (consensus sequence YSPTSPS), serine 2 and serine 5. CTD-serine 5 phosphorylation is a hallmark of promoter-bound RNAP II, whereas CTD-serine 2 phosphorylation indicates transcriptional elongation (46). Both BUNV and LACV were shown to preferentially affect CTD-serine 2 phosphorylation, indicating specific inhibition of host mRNA elongation (28, 44).

**FIG 1:**
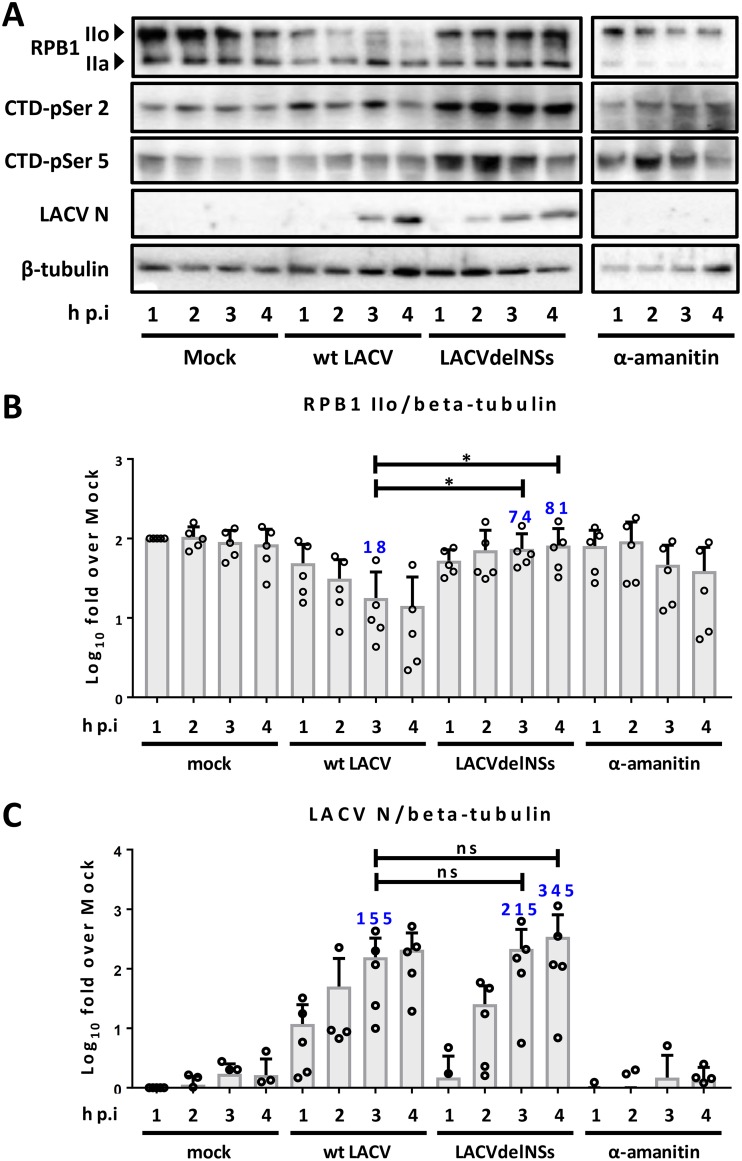
Rapid and specific RPB1 degradation by wt LACV. HuH-7 cells were infected with wt LACV or LACVdelNSs viruses (MOI of 10) or treated with α-amanitin (10 μg/ml). (A) Immunoblot analyses for the various RPB1 states and for viral N at 1 to 4 h p.i., using the antibodies indicated on the left. Immunoblot signals of RPB1 IIo (B) and LACV N (C) were quantified and normalized to the tubulin signal. Means, SD, and individual data points from five independent experiments are shown. A nonparametric, one-tailed Wilcoxon’s paired signed-rank test was applied to test for a potential difference in signals for RPB1 IIo and LACV N between wt LACV 3-h p.i. and the LACVdelNSs 3 and 4-h p.i. time points. *, *P* < 0.05; ns, nonsignificant. RT-qPCR analyses confirmed the similar replication rates and showed that comparable amounts of input virus were used in all cases (data not shown).

Previously, we demonstrated the degradation of the hyperphosphorylated RNAP IIo by NS-expressing wt LACV in a time course experiment that started at 5 h postinfection (p.i.) (28). Meanwhile, however, it has become clear that the host cell elicits IFN induction even earlier, upon detection of the nucleocapsid-borne double-stranded RNA panhandle by RIG-I immediately after virus entry (32). Moreover, reverse transcription-quantitative PCR (RT-qPCR) analyses demonstrated that mRNA transcription by LACV is detectable as early as 1 h p.i. (data not shown). To address this immediate early step of infection, we conducted a time course experiment in human HuH-7 cells (infected at a multiplicity of infection [MOI] of 10) that covered 1 to 4 h p.i. Figure 1A shows that even at 1 h p.i. there is a reduced RPB1 IIo signal in wild-type (wt) LACV-infected cells, whereas infection with an NSs-deleted LACV (LACVdelNSs) had no such effect. These differences in RPB1 IIo levels are not due to potential differences in viral replication, as demonstrated by comparisons of the immunoblot signal quantifications for RPB1 IIo and LACV N at 3 and 4 h p.i. (Fig. 1B and C). Curiously, the phosphorylation states of either RPB1 CTD-serine 2 or 5 are not severely diminished at these time points (in contrast to longer infections [28, 44]), probably due to short-term activation under infection, as seen for the delNSs virus. Interestingly, the application of α-amanitin, a pharmaceutical transcription inhibitor known to induce RPB1 degradation (48), showed a slightly reduced effect than wt LACV. α-Amanitin was given at the same time as the virus, but unlike NSs, which first has to be expressed, the full inhibitor dose is present right from the start. Similar results were obtained with the global inhibitor of DNA-dependent RNA polymerases, actinomycin D (data not shown). The finding that LACV NSs is on a par with a chemical RNAP II inhibitor underscores the surprisingly fast and efficient destruction of RPB1 by LACV NSs.

**FIG 2:**
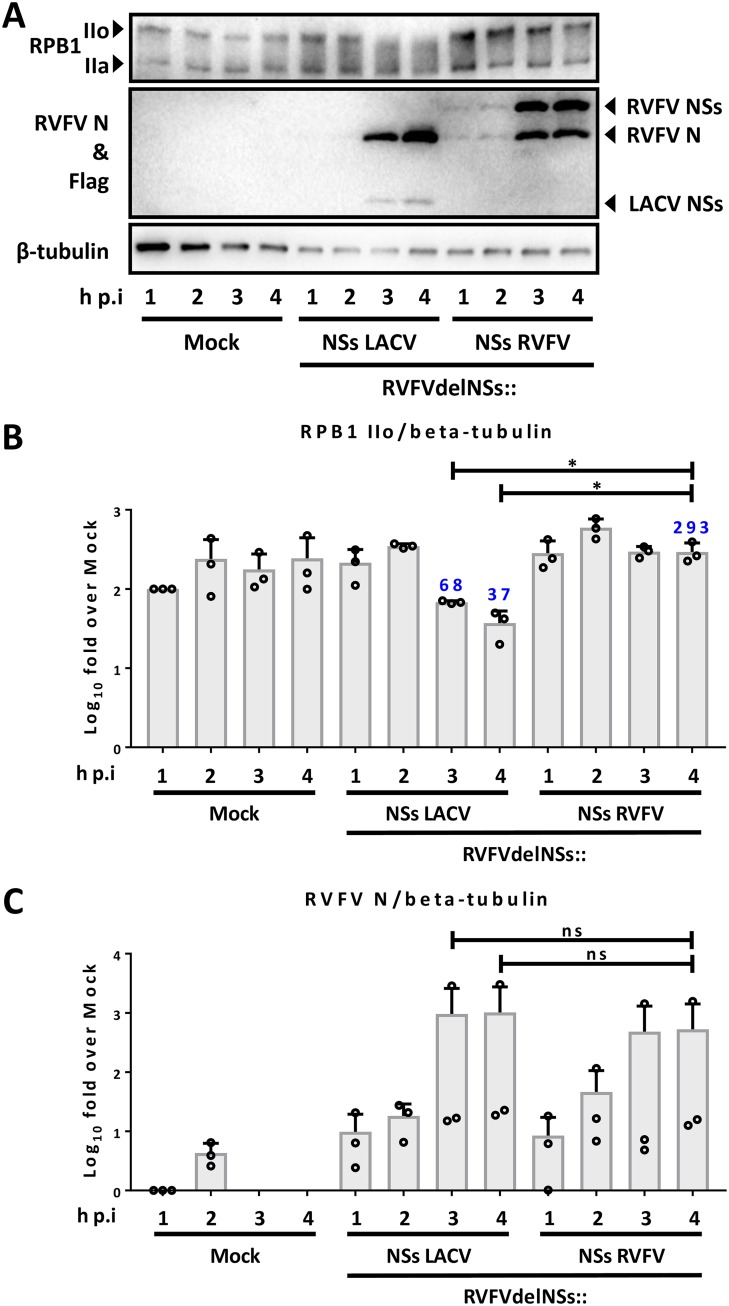
RPB1 degradation by LACV NSs expressed by RVFV. HuH-7 cells were infected at an MOI of 10 with recombinant RVFV expressing either LACV NSs (rRVFVΔNSs::Flag-NSs_LACV_) or RVFV NSs (rRVFVΔNSs::Flag-NSs_RVFV_), and samples for immunoblotting were taken at the indicated times p.i. (B and C) Means, SD, and individual data points from three independent experiments, analyzed as described for Fig. 1.

Besides LACV and related orthobunyaviruses, also Rift Valley fever virus (RVFV; genus *Phlebovirus*, family *Phenuiviridae*, order *Bunyavirales*) encodes an NSs that strongly inhibits RNAP II activity (49, 50). The two NSs proteins play the same biological role (IFN induction antagonism) and target the same cellular function (RNAP II mRNA transcription) but are unrelated with respect to size and amino acid sequence. Using recombinant RVFV encoding the NSs genes of either LACV or RVFV (both equipped with a Flag tag), we compared their RPB1-destructive activities, again in HuH-7 cells within the first 4 h of infection at an MOI of 10. As shown in Fig. 2, the NSs of RVFV is expressed from 1 h p.i. on, but exhibited no RNAP II-destructive activity. RVFV-expressed LACV NSs, in contrast, becomes detectable at 3 h p.i. and diminishes RNAP IIo from this time point on. Thus, taken together, our data show an astonishingly rapid attack of RNAP II by LACV NSs, with an efficiency comparable to a pharmaceutical transcription blocker. Moreover, the RNAP II inhibition profile is different from the other established bunyaviral RNAP II inhibitor, RVFV NSs.

### The Elongin complex is involved in NSs-mediated RNAP II suppression and IFN antagonism.

**FIG 3:**
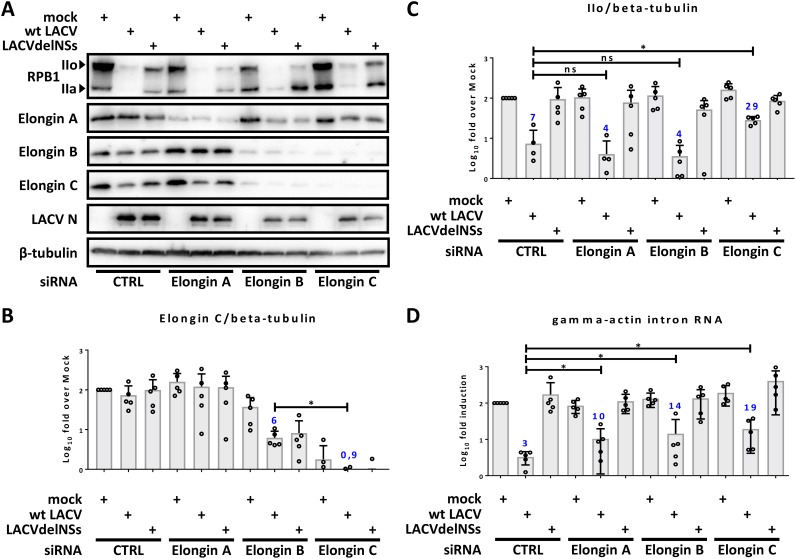
Elongin C and RPB1 degradation. A549 cells were transfected with siRNAs against the mRNAs for Elongin A, B, or C or a negative control (CTRL). (A to D) Cells were mock infected or infected with wt LACV or LACVdelNSs (MOI of 10) for 16 h. (A and B) The presence of RPB1, Elongin A/B/C, LACV N, or β-tubulin was monitored by immunoblotting (A), and the bands of Elongin C and RPB1 IIo were normalized to β-tubulin (B and C). (D) Amounts of γ-actin intron RNA were measured by RT-qPCR. (B to D) Means SD, and individual data points from five independent experiments, analyzed as described for Fig. 1.

The phenotype of RNAP II degradation by LACV NSs resembles the one occurring after DNA damage (28). When RNAP II translocation on DNA is stalled by genotoxic damages or chemical inhibitors such as α-amanitin (46), the RPB1 subunit becomes ubiquitinated and proteasomally degraded (51). The Elongin E3 ubiquitin ligase complex, consisting of the subunits Elongin A, B, and C, was shown to be involved in degradative polyubiquitination of mammalian RPB1 (52–55). In undamaged cells, however, Elongin A/B/C promotes RNAP II transcription (hence its name) by decreasing transient RNAP II pausing (56). We investigated a possible involvement of the Elongin complex in NSs action. First of all, we knocked down mRNAs of the individual Elongins and monitored the effect under LACV infection. Human A459 cells were transfected with siRNA against Elongin A, B, or C; infected with wt LACV or LACVdelNSs (MOI of 10); and then lysed and immunoblotted 16 h later. Figure 3A demonstrates that the small interfering RNAs (siRNAs) were strongly reducing the individual protein levels and that Elongins B and C seem to stabilize each other, since the knockdown of either of these reduced levels of the other one. However, quantification of the immunoblot signals show that Elongin C is most reduced when knocked down by specific siRNA, whereas under conditions of Elongin B knockdown residual amounts of Elongin C remain (Fig. 3B). Regarding RNAP, the levels of the IIo fraction are still largely suppressed by LACV infection when Elongins were depleted by siRNAs (see Fig. 3A). However, quantification and statistics of the immunoblot signals revealed that RPB1 IIo levels partially recovered under conditions of Elongin C knockdown (Fig. 3C). Elongin A or B knockdown, in contrast, had no such effect.

We also measured another hallmark of NSs action on RNAP II, the suppression of γ-actin intron RNA levels. Introns have a short half-life (57), and blockade of RNAP II elongation by LACV NSs dries out their supply within 4 h of infection (28). As shown in Fig. 3D, wt LACV infection suppressed γ-actin intron RNA down to approximately 3% of mock levels. Interestingly, siRNA-mediated removals of either Elongin A, B, or C were all able to rescue γ-actin intron RNA significantly and to a certain extent, but again the Elongin C knockdown reached the highest level. Compared to the control siRNA, Elongin C siRNA alleviated the wt LACV-triggered γ-actin intron RNA reduction from 3 to 19% of the mock levels, i.e., by a factor of ∼6.

**FIG 4:**
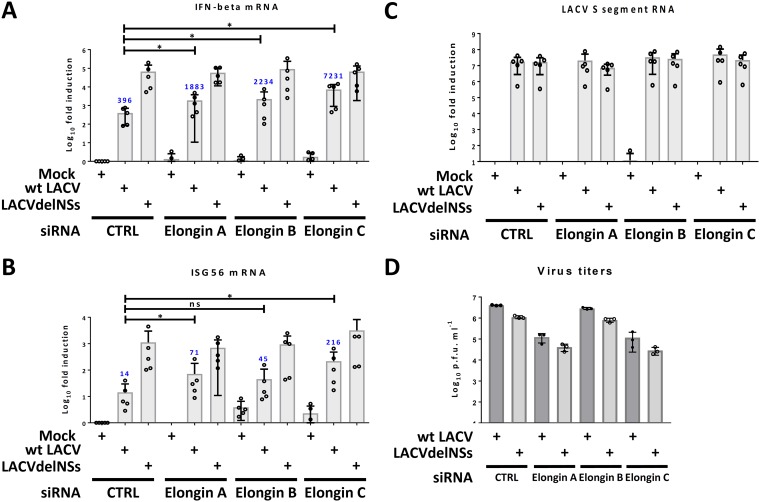
Elongin C and IFN induction. The RNA samples used for Fig. 3E were also analyzed for the changes in mRNAs for IFN-β (A), ISG56 (B), and LACV S segment (C). (D) A549 cells were infected with the respective viruses (MOI of 0.01), and titers were measured at 24 h p.i.

The biologically relevant reason for LACV NSs to degrade transcribing RNAP II is suppression of IFN induction at the mRNA transcription level (35). When expression of the three Elongins in A549 cells was individually suppressed by siRNA transfection and IFN induction was measured, a similar picture as with the γ-actin intron RNA reduction emerged. Suppression of Elongin C rescued IFN-β mRNA induction in wt LACV-infected cells from ∼400-fold to >7,000-fold (compared to the approximately 70,000-fold measured for the delNSs virus), i.e., by more than 1 log_10_ (Fig. 4A). siRNAs against Elongin A or B, in contrast, rescued IFN-β induction in wt LACV-infected cells at a lower, but still significant level. ISG56 (IFIT1) is another virus-induced gene that plays a role in the immediate early antiviral state (58). Also, ISG56 mRNA induction was rescued best in wt LACV-infected cells when Elongin C expression was impeded (Fig. 4B). Importantly, under these single-step growth conditions (MOI of 10, 16 h of infection) none of the Elongin knockdowns significantly influenced viral RNA levels, neither of wt LACV nor of LACVdelNSs (Fig. 4C). However, since some siRNAs did increase IFN and ISG56 mRNA levels in wt LACV-infected cells, we also tested the effect of the Elongin knockdowns in the IFN-competent A549 cells under multistep growth conditions (MOI of 0.01), at an extended incubation time (24 h) and with virus titrations. Indeed, with this more sensitive setting we observed the expected difference between wt LACV and the LACVdelNSs virus (35) and that the knockdown of Elongins A or C (but not B) reduced the growth of both wt LACV and the LACVdelNSs virus (Fig. 4D). Thus, again Elongin C turned out to be a factor exhibiting a clear impact on LACV.

Taken together, our siRNA experiments establish the Elongin A/B/C complex, and especially Elongin C, as a contributor to RPB1 degradation, RNAP II transcription shutoff, and IFN mRNA downregulation by LACV NSs.

### LACV NSs relocalizes Elongin C.

**FIG 5:**
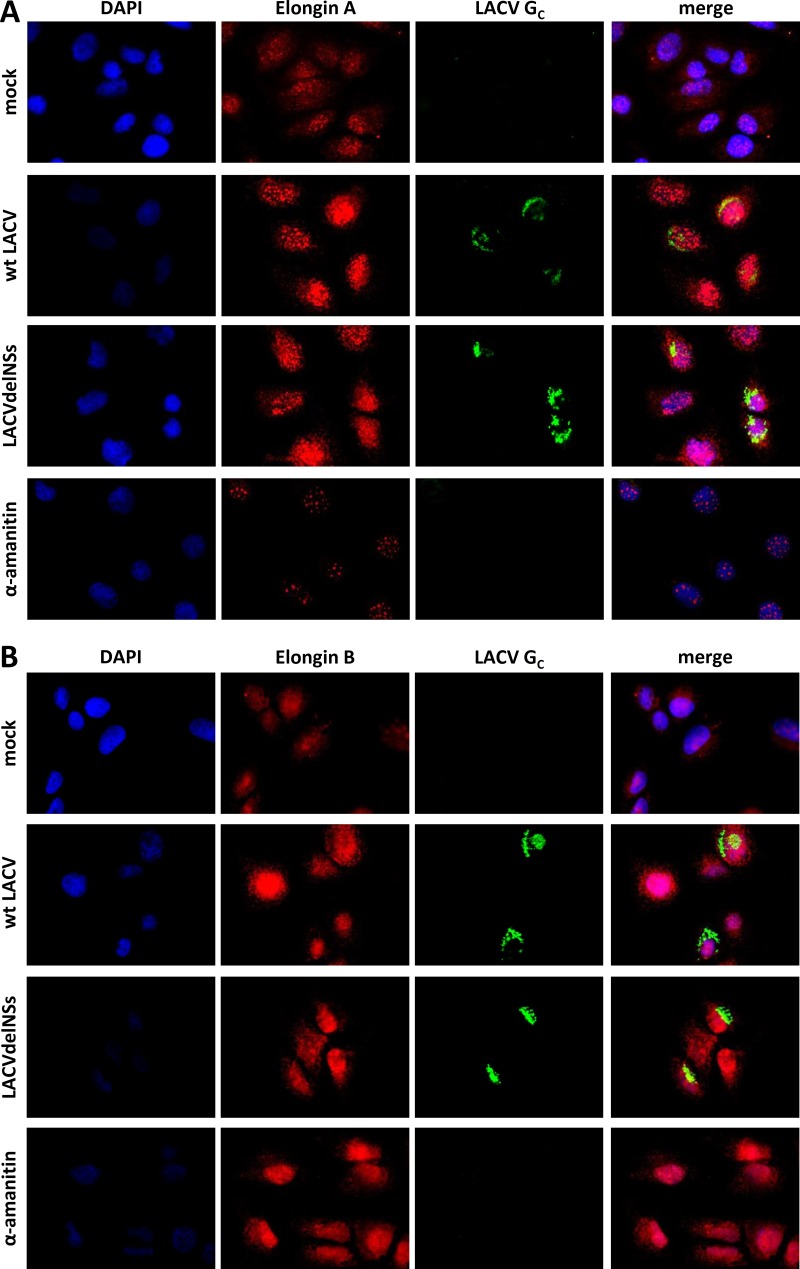
Subcellular localization of Elongin A and B. HuH-7 cells were either mock infected, infected with wt LACV or LACVdelNSs (MOI of 1), or treated with α-amanitin (10 μg/ml) for 16 h. The cells were fixed, permeabilized, and then stained against endogenous Elongin A (A) or Elongin B (B) and LACV Gc protein; the nucleus was counterstained with DAPI.

**FIG 6:**
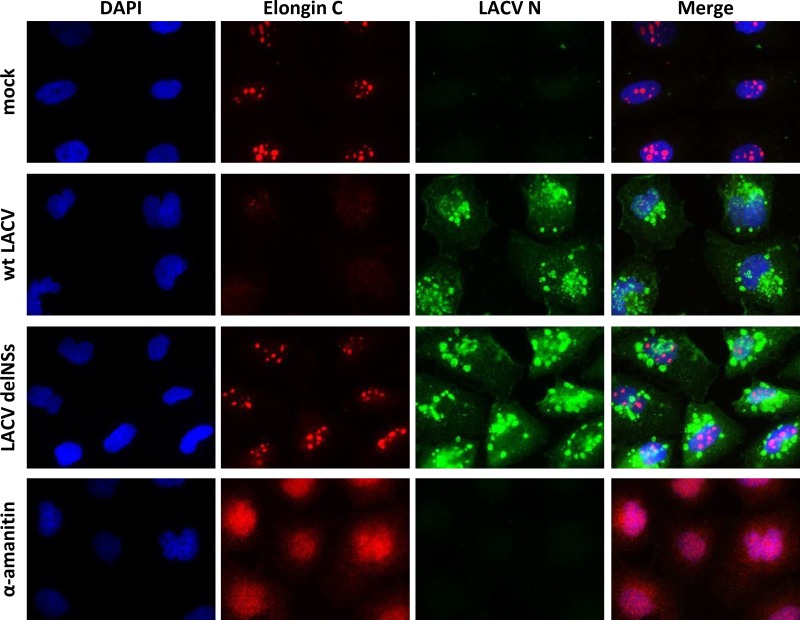
Subcellular localization of Elongin C. HuH-7 cells were infected or α-amanitin treated as indicated for Fig. 5 and then processed for immunofluorescence to stain against endogenous Elongin C and the LACV N protein.

Using immunofluorescence analysis, we sought to observe the behavior of the Elongins in LACV-infected cells. To better distinguish between primary effects caused by NSs and possible secondary effects caused by the NSs-mediated RPB1 degradation and block in host mRNA transcription, we treated the HuH-7 cells in parallel with the RNAP II inhibitor α-amanitin. In the immunofluorescence experiments, Elongin A exhibited in uninfected cells a nuclear signal with the tendency to form speckles (Fig. 5A). The nuclear pattern did not change in infected cells (MOI of 1), independent of whether it was wt LACV or LACVdelNSs. The Elongin A speckles, however, diminished in signal strength and number when α-amanitin was applied. Elongin B produced a nuclear immunofluorescence signal that did not change by any of the treatments (Fig. 5B). Elongin C also exhibited a nuclear speckle pattern in uninfected cells and in cells infected with the LACVdelNSs virus (Fig. 6). In cells infected with wt LACV, however, the speckles were barely visible, whereas under α-amanitin treatment the speckles dissolved but the signal remained nuclear. Since on the other hand protein levels of all Elongins remained unchanged under infection (see Fig. 3), the diminished Elongin C signal in wt LACV-infected cells apparently derived from a relocalization and dilution throughout the cell.

Thus, infection with the NSs-expressing wt LACV converted the nuclear Elongin C speckles in a manner that was unique and clearly distinct from α-amanitin. The subcellular distribution of elongin A and B, in contrast, remained unchanged by infection, although α-amanitin had an influence on Elongin A distribution. From the different phenotypes of infection and α-amanitin we conclude that the redistribution of Elongin C is specific for LACV NSs and not simply a consequence of its ability to block RNAP II.

### Elongin C relocalization is a secondary effect of NSs action.

**FIG 7:**
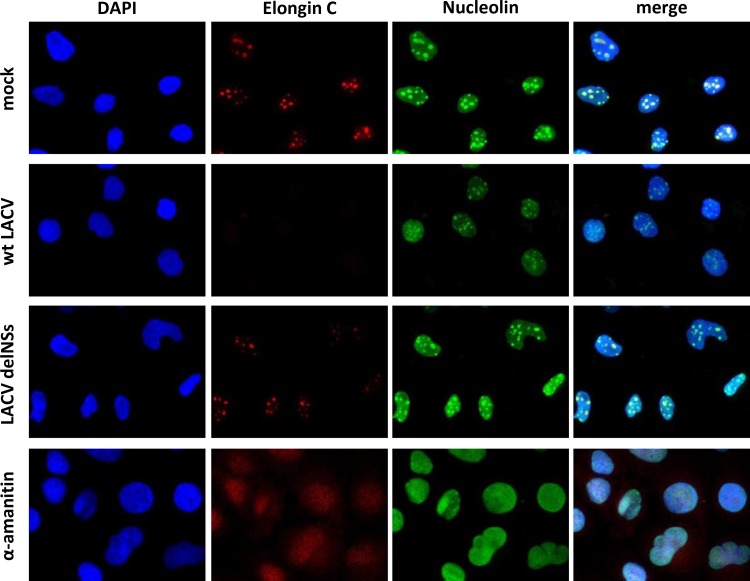
Effect of LACV NSs on nucleolin. HuH-7 cells were infected or α-amanitin treated as indicated for Fig. 5 and immunostained against endogenous Elongin C and nucleolin. The nucleus was counterstained with DAPI.

The subnuclear distribution pattern of Elongin C in uninfected cells is reminiscent of nucleoli, the sites of rRNA synthesis by RNAP I and ribosome biogenesis (59). Indeed, using nucleolin as marker, we detected a clear and 1:1 overlap with the Elongin C signal in HuH-7 cells (Fig. 7). Both nucleolin and Elongin C remained unchanged in LACVdelNSs-infected cells but became evenly distributed in the nucleus upon α-amanitin treatment. wt LACV-infected cells (MOI of 1) exhibited an intermediate phenotype of nucleolin distribution, with still distinguishable but weaker nucleolar speckles, whereas the Elongin C signal became undetectable, as shown above. These results are in line with our previous results showing that LACV NSs impedes neither RNAP I transcription nor nucleolar localization of RNAP I transcripts (28).

**FIG 8:**
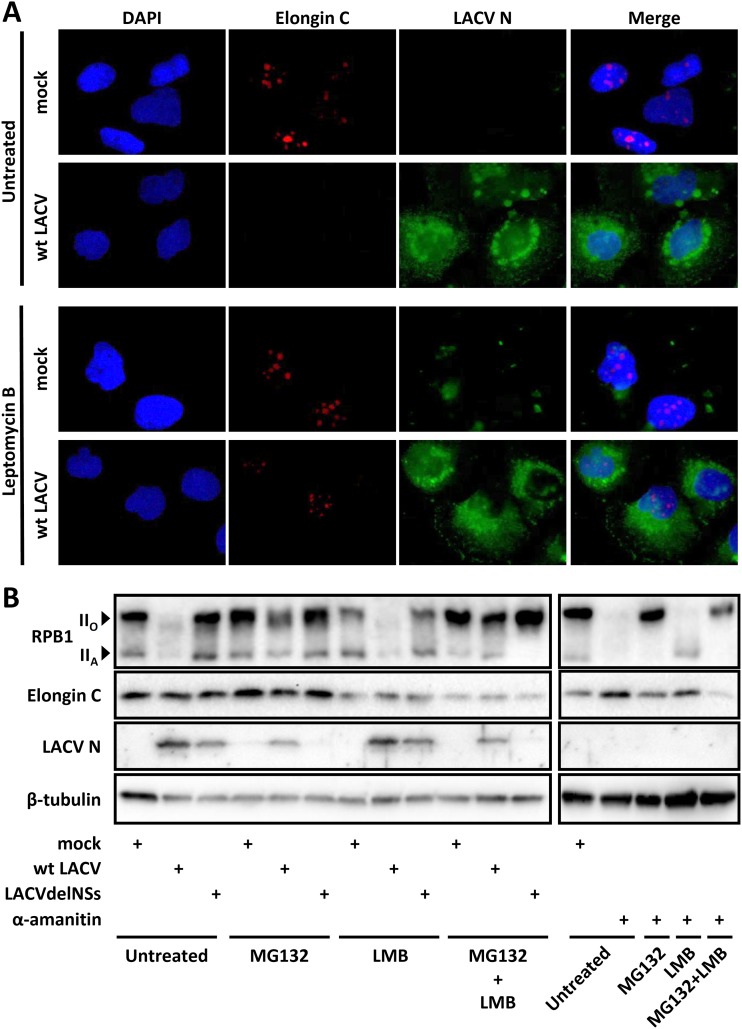
NSs-driven Elongin C redistribution and nuclear export. (A) Immunofluorescence analysis. HuH-7 cells were pretreated with LMB (16 nM) for 1 h and then infected with wt LACV (MOI of 1). Untreated and mock infection controls were done in parallel. At 16 h p.i., the cells were immunostained against Elongin C and LACV N, and the nucleus was counterstained with DAPI. (B) Immunoblot analysis. HuH-7 cells were pretreated with LMB as in panel A and then infected with wt LACV or LACVdel (MOI of 10) or treated with α-amanitin. After removal of the inoculum 1 h later, medium was added containing either MG132 (10 μM) or LMB (16 nM) or a combination of the two. Untreated and mock-infected controls were evaluated in parallel. At 8 h p.i., the samples were analyzed by immunoblotting with the indicated antisera.

NSs seems to specifically disassemble the nucleolar Elongin C speckles without entirely destroying the structural integrity of the nucleoli. Since under NSs on one hand Elongin C becomes undetectable in immunofluorescence but, on the other hand, the levels on immunoblots remain unchanged, we investigated the possibility of a redistribution to the cytoplasm. Leptomycin B (LMB) is an inhibitor of CRM1/exportin 1, the major nuclear export factor for proteins. Using LMB, we could indeed trap Elongin C in the nucleoli of HuH-7 cells infected with wt LACV at an MOI of 1 (although the signal was somewhat weaker than in mock infection) (Fig. 8A). This confirms our assumption of an NSs-driven redistribution out of the nucleus, either by expulsion or by cytoplasmic retention. Nonetheless, immunoblots of HuH-7 cells infected with viruses at an MOI of 10 show that blocking nuclear export by LMB could not relieve the destruction of RPB1 by NSs, in contrast to the proteasomal inhibitor MG132 (Fig. 8B). Also, when α-amanitin was applied we found that LMB was unable to entirely rescue RPB1 from proteasomal destruction.

LMB had no impact on wt LACV titers (data not shown) or the N protein expression (see Fig. 8B). Thus, NSs removes Elongin C from the nucleus in a Crm1-dependent manner, but this mechanism seems a secondary phenomenon rather than an essential step in the attack of RNAP II. Altogether, our data show that the E3 ubiquitin ligase subunit Elongin C is involved in the NSs-mediated destruction of the RNAP II large subunit RPB1 and the suppression of IFN induction, and gets relocalized as a consequence.

## DISCUSSION

Morphological changes of nucleoli were long known as a hallmark of orthobunyavirus infection (60). Moreover, recently a nucleolar localization sequence important for NSs action was described for the orthobunyavirus SBV (45). Our findings that Elongin C (i) colocalizes with nucleolin in uninfected cells, (ii) contributes to the NSs-mediated RNAP II degradation, and (iii) becomes redistributed in the presence of NSs are in line with those earlier observations. Thus, Elongin C appears to be a player in the anti-IFN strategy of LACV, a view further underscored by the increase in IFN induction and RNAP II activity by> log_10_ step in Elongin C-deficient cells that were infected with wt LACV.

The cellular Elongin complex consisting of Elongin A, B, and C is able to foster both the elongation of mRNA transcription by RNAP II (56) and (the B/C complex) the degradation of RPB1 under conditions of stress (53–55). These activities are congruent with the action of orthobunyavirus NSs, which targets exactly these activities, as it inhibits mRNA elongation and drives RPB1 degradation (28, 44). Moreover, the fact that mostly Elongin C participated in these NSs activities is in accord with our immunofluorescence data which show its relocalization by NSs but not that of Elongin A and B. Moreover, Elongin C was the only Elongin complex subunit that localizes to the nucleoli, which in turn are reorganized by orthobunyaviral NSs proteins (45, 60).

Our attempts to robustly demonstrate a direct interaction between LACV NSs and Elongin C in transfected cells have failed (data not shown), in line with the absence of Elongin C in the LACV NSs interactome (61). Moreover, LACV NSs does not exhibit a discernible nucleolar localization but is faintly distributed all over the nucleus (28), and the nucleolar localization sequence detected for SBV NSs is not conserved in LACV NSs (45). Thus, the effect of NSs on Elongin C in the nucleoli is most likely transient or indirect. Also, the relocation of Elongin C might be a secondary effect and not causative, since nuclear export inhibition by LMB partially prevented Elongin C redistribution but not RPB1 degradation by NSs. We therefore hypothesize that LACV NSs recruits an Elongin C-dependent host cell pathway that leads to RPB1 degradation and inhibition of host mRNA transcription. The relocalization of Elongin C is a morphological consequence of this process (but NSs specific, since RNAP II inhibition by α-amanitin has a different phenotype), but it is not necessarily causative. Why Elongin A and B seem less important remains unclear. A slight but statistically significant rescue of RNAP II transcription activity was, however, measurable also in those knockdowns.

Despite the significant impact of the Elongin C knockdown, it was not sufficient to entirely rescue RNAP II and IFN induction from NSs-mediated destruction and blockade. This is comparable to the observation with RVFV NSs and its cellular cofactor, the E3 ubiquitin ligase subunit FBXO3. Similar to the situation with LACV NSs and Elongin C, IFN induction by wt RVFV is rescued by one log_10_ step in cells depleted of FBXO3, but an NSs-deleted virus mutant still induced IFN-β by another log_10_ step (62). RVFV NSs is known to counteract IFN induction by several mechanisms, namely, (i) sequestration of subunits p44 and XPB of the general RNAP II transcription factor TFIIH, (ii) destruction of the TFIIH subunit p62 (mediated by mentioned FBXO3), and (iii) recruitment of the IFN promoter suppressor SAP30 (49, 50). It is therefore conceivable that LACV NSs, despite having approximately just one-third the size of RVFV NSs, has similarly evolved several independent mechanisms to destroy RNAP II and suppress IFN induction and that the Elongin C-dependent branch is just one of them. Removing Elongin C can help to uncover these additional anti-IFN strategies which may contribute to the degradation of RPB1 and breakdown of RNAP II activity, which is even faster than for RVFV NSs.

An interesting side observation of our studies was the establishment of Elongin C (but not A or B) as a nucleolar protein. We were able to locate Elongin A in a database of the nucleolar proteome (63) but not Elongin C. We therefore hypothesize a transient and weak association, which is probably disrupted upon cell lysis or nucleoli preparation. In support of this, LACV (and SBV) NSs disturbs the nucleolar organization only slightly (and LACV NSs still allows RNAP I activity there [28]), but this might be sufficient to release Elongin C. α-Amanitin, in contrast, dissolves nucleoli entirely and also redistributes Elongin C.

In summary, we have shown that LACV NSs triggers the destruction of RNAP II in a surprisingly fast manner, starting at 1 h p.i. This is in line with the earlier observation that the pattern recognition receptor RIG-I of the IFN immune system is capable of recognizing already the incoming LACV nucleocapsids, thus requiring an immediate viral counterreaction (32). The fast RNAP II degradation by NSs leads to general blockade of host mRNA production and hence to an inability of the cell to produce IFN and alert the surrounding cells. RNAP II degradation is due to ubiquitination and proteasomal activity and involves the host factor Elongin C that is expelled from the nucleoli during the course of these events. This fast and efficient way of shutting down the IFN response contributes to the virulence of LACV, and it will be interesting to see whether other pathogenic orthobunyaviruses are using the same mechanism.

## MATERIALS AND METHODS

### Cells and reagents.

Human HuH-7 and A549 cells, African green monkey Vero 76 cells, and CV-1 cells were maintained in Dulbecco modified Eagle medium (Gibco, catalog no. 21969035) supplemented with 10% fetal calf serum, 2 mM l-glutamine (Gibco, catalog no. 25030081), and 50 U/ml penicillin and 50 μg/ml streptomycin (Gibco, catalog no. 15140122). The specific use of the respective cell line is indicated in the figure legends. The RPB1-specific inhibitor α-amanitin was purchased from AppliChem (A1485,0001), the nuclear export inhibitor leptomycin B was purchased from US Biological (L1671-38B), and the proteasomal inhibitor MG132 was purchased from Cayman Chemical.

### Viruses and virus titration.

Recombinant wild-type La Crosse virus (wt LACV) and LACV lacking NSs expression (LACVdelNSs) were rescued as described previously (64). Recombinant Rift Valley fever virus expressing a C-terminal 3×Flag tag NSs (rRVFVΔNSs::Flag-NSs_RVFV_) or a N-terminal 3×Flag tag LACV NSs (rRVFVΔNSs::Flag-NSs_LACV_) was rescued as described previously (65).

Both LACV (BSL-2) and RVFV (BSL-3) were propagated on Vero 76 cells by infection at an MOI of 0.001 with the respective virus and cultivating the cells for 3 days. The supernatant was cleared by centrifugation, and the virus titer was determined by plaque test on CV-1 cells, using an Avicel overlay (66). After 3 days of incubation, the overlay was washed of using phosphate-buffered saline (PBS), and the cell layer was stained with staining solution (0.75% crystal violet, 3.75% formaldehyde, 20% ethanol, 1% methanol) for 10 min. The amount of virus was then calculated as PFU/ml.

### Immunoblot analysis.

After the cells were washed once with PBS, they were lysed in a 1:1 mixture of tissue protein extraction reagent (Thermo Scientific, catalog no. 78510) supplemented with phosphatase (Calbiochem, catalog no. 524625) and protease (Roche, catalog no. 4693116001) inhibitors and 2× sample buffer (62.5 mM Tris from a 0.5 M stock [pH 6.8], 25% glycerol, 2% sodium dodecyl sulfate [SDS], 0.01% bromophenol blue, and 5% 2-mercaptoethanol). The lysates were boiled for 10 min, separated by SDS-PAGE, and then blotted onto polyvinylidene difluoride membranes. The membranes were blocked with 5% (wt/vol) nonfat dry milk powder in TBS-T and probed with primary antibodies against the following targets: RPB1/Pol II (N-20; Santa Cruz, catalog no. sc-899, 1:500, rabbit, polyclonal), RPB1 CTD-pSer 2/Ser2 α-CTD (3E10; 1:500, rat, polyclonal), RPB1 CTD-pSer 5/Ser5 α-CTD (3E8 [both antibodies were kindly provided by Dirk Eick, Helmholtz-Zentrum Munich, Germany], 1:500, rat, polyclonal), anti-LACV N (kindly provided by Georg Kochs, Institute of Virology, Freiburg, Germany; 1:15,000, rabbit, polyclonal), anti-RVFV N (67; 1:2,000, mouse, polyclonal), Elongin A/TCEB3/SIII/p110 (110 kDa; Sigma-Aldrich, catalog no. HPA005910, 1:500, rabbit, polyclonal), Elongin B (18 kDa; Santa Cruz, sc-11447, 1:250, rabbit, polyclonal), Elongin C/SIII p15 (15 kDa; BD Transduction Laboratories, catalog no. 610761, 1:250, mouse, monoclonal), anti-β-tubulin (Abcam, ab6046, 1:1,000, rabbit, polyclonal), and anti-Flag (TM; Sigma-Aldrich, catalog no. F7425, 1:3200, rabbit, polyclonal). The secondary antibodies were peroxidase-conjugated anti-mouse IgG (Thermo Fisher, catalog no. 31430, 1:40,000, goat, polyclonal), peroxidase-conjugated anti-rabbit IgG (Thermo Fisher, catalog no. 31460, 1:40,000, goat, polyclonal), and peroxidase-conjugated anti-rat IgG (Jackson ImmunoResearch, catalog no. 712-036-150, 1:40,000, donkey, polyclonal). Western blot signals were detected on a Chemidoc (Bio-Rad) using SuperSignal West Femto maximum sensitivity substrate (Thermo Scientific, catalog no. 34096).

### Densitometry analysis.

Western blot signals were quantified using the Image Lab software provided with the Chemidoc machine (Bio-Rad). The intensities of the respective bands were measured using the volume tool, and the adjusted volume, accounting for the background, was used for each band. The bands of interest were first normalized to the loading control (β-tubulin) of the respective sample, and the normalized values were then compared to mock-treated samples of the same experiment. The graphs presented depict fold changes compared to mock-treated samples, with mock values set to 100%.

### siRNA knockdown of the Elongin complex components.

Knockdowns of the indicated genes were performed twice by reverse transfection of siRNAs. All siRNAs were purchased from Qiagen: AllStar negative control siRNA (1027280), Elongin A (GeneSolution GS6924), Elongin B (GeneSolution GS6923), and Elongin C (GeneSolution GS6921). GeneSolution consists of four validated siRNAs against the respective transcript that were mixed in equimolar ratios with each other. A final concentration of 50 nM for each siRNA solution was reverse transfected using Lipofectamine RNAiMAX (Invitrogen, catalog no. 13778075) according to the manufacturer’s instructions. After 4 h, the medium was changed, and the cells were incubated for 3 days. One day before the experiment, the cells were harvested and counted, and equal amounts of the cells were again reverse transfected as described above.

### Real-time RT-PCR.

Total cellular RNA was isolated with an RNeasy minikit (Qiagen, catalog no. 74104) eluted in 30 μl of sterile H_2_O, and 100 ng of RNA of each sample was used as a template. cDNA synthesis was random primed by using a QuantiTect reverse transcription kit (Qiagen, catalog no. 205313). For PCR, a QuantiTect SYBR green PCR kit (Qiagen catalog no. 204143l; random-primed cDNA) or SYBR Premix *Ex Taq* (Tli RNase H Plus; TaKaRa catalog no. RR420A; strand-specific cDNA) was used. Primers were either purchased from Qiagen (IFN-β, QT00203763; IFIT1/ISG56, QT00201012; 18S RNA, QT00199367) or custom ordered for γ-actin intron 3 (forward, 5′-GCTGTTCCAGGCTCTGTTCC-3′; reverse, 5′-ATGCTCACACGCCACAACATGC-3′) (68) and LACV S segment (forward, 5′-GGGTATATGGACTTCTGTG-3′; reverse, 5′-GCCTTCCTCTCTGGCTTA-3′). The qPCR was carried out in a StepOne real-time PCR system (Applied Biosystems). All values obtained were normalized against the 18S RNA signal by using the ΔΔ*C_T_* method (69).

### Immunofluorescence analysis.

Cells, grown on coverslips to 30 to 50% confluence, were either mock treated, infected with wt LACV or LACVdelNSs, or treated with α-amanitin for the indicated times. After the cells were fixed with 3% paraformaldehyde, they were permeabilized with 0.5% Triton X-100 in PBS. Unspecific staining was reduced by incubating the cells in blocking and staining solution (2% bovine serum albumin, 5% glycerol, and 0.2% Tween 20 in PBS) for 30 min at room temperature. The following primary antibodies, diluted in blocking and staining solution, were used: rabbit polyclonal anti-LACV N (1:500), mouse monoclonal anti-LACV G_C_ (1:400, kindly provided by Francesco Gonzales-Scarano, Perelman School of Medicine, Philadelphia, PA), Elongin A (1:500, rabbit; Sigma-Aldrich, HPA005910), Elongin B (1:100, rabbit; Santa Cruz, sc-11447), Elongin C (1:250, mouse; BD Transduction Laboratories, 610761), and nucleolin (1:1,000; Abcam, ab22758). The primary antibodies were incubated on cells at room temperature for 1 h, followed by three washes in PBS. The secondary antibody was either Alexa Fluor 488 donkey anti-mouse IgG (1:200; Invitrogen/Molecular Probes, A21202), Alexa Fluor 555 Donkey anti-mouse IgG (1:200, Invitrogen/Molecular Probes, A31570), Alexa Fluor 555 donkey anti-rabbit IgG (1:200; Invitrogen/Molecular Probes, A31572), or Alexa Fluor 488 donkey anti-rabbit IgG (1:200; Invitrogen/Molecular Probes, A21206) used. The nuclei were visualized by staining the chromatin with DAPI (4′,6′-diamidino-2-phenylindole; 1:100) at the same time as the secondary antibody incubation. After 45 min of incubation, the cells were washed again three times in PBS and once in H_2_O and then mounted using Fluorsave solution (Calbiochem). Images were taken with an Apotome (Zeiss) at a magnification of ×63.

### Statistical analysis.

The quantitative data are presented as means ± the standard deviations (SD) for three biological replicates. The statistical significance between two groups was evaluated by using a nonparametric, one-tailed Wilcoxon paired signed-rank test, with a *P* value of <0.05 considered statistically significant.

## References

[1] Dilcher M, Sall AA, Hufert FT, Weidmann M 2013 Clarifying Bunyamwera virus riddles of the past. Virus Genes 47:160–163. doi:10.1007/s11262-013-0918-y. 23686694

[2] Gerrard SR, Li L, Barrett AD, Nichol ST 2004 Ngari virus is a Bunyamwera virus reassortant that can be associated with large outbreaks of hemorrhagic fever in Africa. J Virol 78:8922–8926. doi:10.1128/JVI.78.16.8922-8926.2004. 15280501 PMC479050

[3] De Regge N 2017 Akabane, Aino, and Schmallenberg virus: where do we stand and what do we know about the role of domestic ruminant hosts and Culicoides vectors in virus transmission and overwintering? Curr Opin Virol 27:15–30. doi:10.1016/j.coviro.2017.10.004. 29096232

[4] Wernike K, Conraths F, Zanella G, Granzow H, Gache K, Schirrmeier H, Valas S, Staubach C, Marianneau P, Kraatz F, Horeth-Bontgen D, Reimann I, Zientara S, Beer M 2014 Schmallenberg virus-two years of experiences. Prev Vet Med 116:423–434. doi:10.1016/j.prevetmed.2014.03.021. 24768435

[5] Sakkas H, Bozidis P, Franks A, Papadopoulou C, Sakkas H, Bozidis P, Franks A, Papadopoulou C 2018 Oropouche fever: a review. Viruses 10:175. doi:10.3390/v10040175. PMC5923469 29617280

[6] Vasconcelos PF, Calisher CH 2016 Emergence of human arboviral diseases in the Americas, 2000–2016. Vector Borne Zoonotic Dis 16:295–301. doi:10.1089/vbz.2016.1952. 26991057

[7] Groseth A, Vine V, Weisend C, Guevara C, Watts D, Russell B, Tesh RB, Ebihara H 2017 Maguari virus associated with human disease. Emerg Infect Dis 23:1325–1331. doi:10.3201/eid2308.161254. 28726602 PMC5547800

[8] Gauci PJ, McAllister J, Mitchell IR, Boyle DB, Bulach DM, Weir RP, Melville LF, Gubala AJ 2015 Genomic characterization of three Mapputta group viruses, a serogroup of Australian and Papua New Guinean bunyaviruses associated with human disease. PLoS One 10:e0116561. doi:10.1371/journal.pone.0116561. 25588016 PMC4294684

[9] Evans AB, Winkler CW, Peterson KE 2019 Differences in neuropathogenesis of encephalitic California serogroup viruses. Emerg Infect Dis 25:728–738. doi:10.3201/eid2504.181016. 30882310 PMC6433036

[10] Harding S, Greig J, Mascarenhas M, Young I, Waddell LA 2018 La Crosse virus: a scoping review of the global evidence. Epidemiol Infect doi:10.1017/S0950268818003096:1-13. PMC6518580 30516125

[11] McJunkin JE, de los Reyes EC, Irazuzta JE, Caceres MJ, Khan RR, Minnich LL, Fu KD, Lovett GD, Tsai T, Thompson A 2001 La Crosse encephalitis in children. N Engl J Med 344:801–807. doi:10.1056/NEJM200103153441103. 11248155

[12] Teleron AL, Rose BK, Williams DM, Kemper SE, McJunkin JE 2016 La Crosse encephalitis: an adult case series. Am J Med 129:881–884. doi:10.1016/j.amjmed.2016.03.021. 27086496

[13] Gaensbauer JT, Lindsey NP, Messacar K, Staples JE, Fischer M 2014 Neuroinvasive arboviral disease in the United States: 2003 to 2012. Pediatrics 134:e642–e650. doi:10.1542/peds.2014-0498. 25113294 PMC5662468

[14] Calisher CH 1994 Medically important arboviruses of the United States and Canada. Clin Microbiol Rev 7:89–116. doi:10.1128/cmr.7.1.89. 8118792 PMC358307

[15] Maes P, Alkhovsky SV, Bào Y, Beer M, Birkhead M, Briese T, Buchmeier MJ, Calisher CH, Charrel RN, Choi IR, Clegg CS, de la Torre JC, Delwart E, DeRisi JL, Di Bello PL, Di Serio F, Digiaro M, Dolja VV, Drosten C, Druciarek TZ, Du J, Ebihara H, Elbeaino T, Gergerich RC, 2018 Taxonomy of the family *Arenaviridae* and the order *Bunyavirales*: update 2018. Arch Virol 163:2295–2310. doi:10.1007/s00705-018-3843-5. 29680923

[16] Elliott RM 2014 Orthobunyaviruses: recent genetic and structural insights. Nat Rev Microbiol 12:673–685. doi:10.1038/nrmicro3332. 25198140

[17] Ferron F, Weber F, de la Torre JC, Reguera J 2017 Transcription and replication mechanisms of *Bunyaviridae* and *Arenaviridae* L proteins. Virus Res 234:118–134. doi:10.1016/j.virusres.2017.01.018. 28137457 PMC7114536

[18] Albornoz A, Hoffmann AB, Lozach P-Y, Tischler ND, Albornoz A, Hoffmann A, Lozach P-Y, Tischler N 2016 Early bunyavirus-host cell interactions. Viruses 8:143. doi:10.3390/v8050143. PMC4885098 27213430

[19] Schoen A, Weber F 2015 Orthobunyaviruses and innate immunity induction: alieNSs versus PredatoRRs. Eur J Cell Biol 94:384–390. doi:10.1016/j.ejcb.2015.06.001. 26095300

[20] Reguera J, Weber F, Cusack S 2010 Bunyaviridae RNA polymerases (L-protein) have an N-terminal, influenza-like endonuclease domain, essential for viral cap-dependent transcription. PLoS Pathog 6:e1001101. doi:10.1371/journal.ppat.1001101. 20862319 PMC2940753

[21] Shi XH, Botting CH, Li P, Niglas M, Brennan B, Shirran SL, Szemiel AM, Elliott RM 2016 Bunyamwera orthobunyavirus glycoprotein precursor is processed by cellular signal peptidase and signal peptide peptidase. Proc Natl Acad Sci U S A 113:8825–8830. doi:10.1073/pnas.1603364113. 27439867 PMC4978261

[22] Shi XH, van Mierlo JT, French A, Elliott RM 2010 Visualizing the replication cycle of bunyamwera orthobunyavirus expressing fluorescent protein-tagged Gc glycoprotein. J Virol 84:8460–8469. doi:10.1128/JVI.00902-10. 20573824 PMC2919021

[23] Eifan S, Schnettler E, Dietrich I, Kohl A, Blomstrom AL 2013 Nonstructural proteins of arthropod-borne bunyaviruses: roles and functions. Viruses (Basel) 5:2447–2468. doi:10.3390/v5102447. PMC3814597 24100888

[24] Sadler AJ, Williams BR 2008 Interferon-inducible antiviral effectors. Nat Rev Immunol 8:559–568. doi:10.1038/nri2314. 18575461 PMC2522268

[25] Schoggins JW, Rice CM 2011 Interferon-stimulated genes and their antiviral effector functions. Curr Opin Virol 1:519–525. doi:10.1016/j.coviro.2011.10.008. 22328912 PMC3274382

[26] Mukherjee P, Woods TA, Moore RA, Peterson KE 2013 Activation of the innate signaling molecule MAVS by bunyavirus infection upregulates the adaptor protein SARM1, leading to neuronal death. Immunity 38:705–716. doi:10.1016/j.immuni.2013.02.013. 23499490 PMC4783152

[27] Taylor KG, Peterson KE 2014 Innate immune response to La Crosse virus infection. J Neurovirol 20:150–156. doi:10.1007/s13365-013-0186-6. 23846288

[28] Verbruggen P, Ruf M, Blakqori G, Overby AK, Heidemann M, Eick D, Weber F 2011 Interferon antagonist NSs of La Crosse virus triggers a DNA damage response-like degradation of transcribing RNA polymerase II. J Biol Chem 286:3681–3692. doi:10.1074/jbc.M110.154799. 21118815 PMC3030371

[29] Kato H, Oh SW, Fujita T 2017 RIG-I-Like receptors and type I interferonopathies. J Interferon Cytokine Res 37:207–213. doi:10.1089/jir.2016.0095. 28475461 PMC5439449

[30] Weber M, Weber F 2014 Segmented negative-strand RNA viruses and RIG-I: divide (your genome) and rule. Curr Opin Microbiol 20:96–102. doi:10.1016/j.mib.2014.05.002. 24930021

[31] Habjan M, Andersson I, Klingstrom J, Schumann M, Martin A, Zimmermann P, Wagner V, Pichlmair A, Schneider U, Muhlberger E, Mirazimi A, Weber F 2008 Processing of genome 5′ termini as a strategy of negative-strand RNA viruses to avoid RIG-I-dependent interferon induction. PLoS One 3:e2032. doi:10.1371/journal.pone.0002032. 18446221 PMC2323571

[32] Weber M, Gawanbacht A, Habjan M, Rang A, Borner C, Schmidt AM, Veitinger S, Jacob R, Devignot S, Kochs G, García-Sastre A, Weber F 2013 Incoming RNA virus nucleocapsids containing a 5′-triphosphorylated genome activate RIG-I and antiviral signaling. Cell Host Microbe 13:336–346. doi:10.1016/j.chom.2013.01.012. 23498958 PMC5515363

[33] Lassig C, Hopfner KP 2017 Discrimination of cytosolic self and non-self RNA by RIG-I-like receptors. J Biol Chem 292:9000–9009. doi:10.1074/jbc.R117.788398. 28411239 PMC5454087

[34] Yoneyama M, Onomoto K, Jogi M, Akaboshi T, Fujita T 2015 Viral RNA detection by RIG-I-like receptors. Curr Opin Immunol 32:48–53. doi:10.1016/j.coi.2014.12.012. 25594890

[35] Blakqori G, Delhaye S, Habjan M, Blair CD, Sanchez-Vargas I, Olson KE, Attarzadeh-Yazdi G, Fragkoudis R, Kohl A, Kalinke U, Weiss S, Michiels T, Staeheli P, Weber F 2007 La Crosse bunyavirus nonstructural protein NSs serves to suppress the type I interferon system of mammalian hosts. J Virol 81:4991–4999. doi:10.1128/JVI.01933-06. 17344298 PMC1900204

[36] Carlton-Smith C, Elliott RM 2012 Viperin, MTAP44, and protein kinase R contribute to the interferon-induced inhibition of Bunyamwera orthobunyavirus replication. J Virol 86:11548–11557. doi:10.1128/JVI.01773-12. 22896602 PMC3486307

[37] Livonesi MC, de Sousa RL, Badra SJ, Figueiredo LT 2007 *In vitro* and *in vivo* studies of the Interferon-alpha action on distinct orthobunyavirus. Antiviral Res 75:121–128. doi:10.1016/j.antiviral.2007.01.158. 17368573 PMC7114330

[38] Tilston-Lunel NL, Acrani GO, Randall RE, Elliott RM 2015 Generation of recombinant Oropouche viruses lacking the nonstructural protein NSm or NSs. J Virol 90:2616–2627. doi:10.1128/JVI.02849-15. 26699638 PMC4810690

[39] Frese M, Kochs G, Feldmann H, Hertkorn C, Haller O 1996 Inhibition of bunyaviruses, phleboviruses, and hantaviruses by human MxA protein. J Virol 70:915–923. doi:10.1128/JVI.70.2.915-923.1996. 8551631 PMC189895

[40] Reichelt M, Stertz S, Krijnse-Locker J, Haller O, Kochs G 2004 Missorting of La Crosse virus nucleocapsid protein by the interferon-induced MxA GTPase involves smooth ER membranes. Traffic 5:772–784. doi:10.1111/j.1600-0854.2004.00219.x. 15355513

[41] Streitenfeld H, Boyd A, Fazakerley JK, Bridgen A, Elliott RM, Weber F 2003 Activation of PKR by Bunyamwera virus is independent of the viral interferon antagonist NSs. J Virol 77:5507–5511. doi:10.1128/jvi.77.9.5507-5511.2003. 12692253 PMC153953

[42] Varela M, Piras IM, Mullan C, Shi X, Tilston-Lunel NL, Pinto RM, Taggart A, Welch SR, Neil SJD, Kreher F, Elliott RM, Palmarini M 2017 Sensitivity to BST-2 restriction correlates with orthobunyavirus host range. Virology 509:121–130. doi:10.1016/j.virol.2017.06.017. 28628828 PMC5526858

[43] Barry G, Varela M, Ratinier M, Blomstrom AL, Caporale M, Seehusen F, Hahn K, Schnettler E, Baumgartner W, Kohl A, Palmarini M 2014 NSs protein of Schmallenberg virus counteracts the antiviral response of the cell by inhibiting its transcriptional machinery. J Gen Virol 95:1640–1646. doi:10.1099/vir.0.065425-0. 24828331 PMC4103064

[44] Thomas D, Blakqori G, Wagner V, Banholzer M, Kessler N, Elliott RM, Haller O, Weber F 2004 Inhibition of RNA polymerase II phosphorylation by a viral interferon antagonist. J Biol Chem 279:31471–31477. doi:10.1074/jbc.M400938200. 15150262

[45] Gouzil J, Fablet A, Lara E, Caignard G, Cochet M, Kundlacz C, Palmarini M, Varela M, Breard E, Sailleau C, Viarouge C, Coulpier M, Zientara S, Vitour D, Gouzil J, Fablet A, Lara E, Caignard G, Cochet M, Kundlacz C, Palmarini M, Varela M, Breard E, Sailleau C, Viarouge C, Coulpier M, Zientara S, Vitour D 2017 Nonstructural protein NSs of Schmallenberg virus is targeted to the nucleolus and induces nucleolar disorganization. J Virol 91:e01263-19. doi:10.1128/JVI.01263-16. PMC5165206 27795408

[46] Chen FX, Smith ER, Shilatifard A 2018 Born to run: control of transcription elongation by RNA polymerase II. Nat Rev Mol Cell Biol 19:464–478. doi:10.1038/s41580-018-0010-5. 29740129

[47] Eick D, Geyer M 2013 The RNA polymerase II carboxy-terminal domain (CTD) code. Chem Rev 113:8456–8490. doi:10.1021/cr400071f. 23952966

[48] Anindya R, Aygun O, Svejstrup JQ 2007 Damage-induced ubiquitylation of human RNA polymerase II by the ubiquitin ligase Nedd4, but not Cockayne syndrome proteins or BRCA1. Mol Cell 28:386–397. doi:10.1016/j.molcel.2007.10.008. 17996703

[49] Ly HJ, Ikegami T 2016 Rift Valley fever virus NSs protein functions and the similarity to other bunyavirus NSs proteins. Virology J 13:118. doi:10.1186/s12985-016-0573-8. 27368371 PMC4930582

[50] Wuerth JD, Weber F, Wuerth J, Weber F 2016 Phleboviruses and the type I interferon response. Viruses-Basel 8:174. doi:10.3390/v8060174. PMC4926194 27338447

[51] Wilson MD, Harreman M, Svejstrup JQ 2013 Ubiquitylation and degradation of elongating RNA polymerase II: the last resort. Biochim Biophys Acta 1829:151–157. doi:10.1016/j.bbagrm.2012.08.002. 22960598

[52] Aso T, Kamura T, Kitajima S, Conaway RC, Conaway JW, Yasukawa T 2009 Mammalian Elongin A complex mediates DNA-damage-induced ubiquitylation and degradation of Rpb1. FASEB J 23. 10.1038/emboj.2008.249 PMC2609743 19037258

[53] Harreman M, Taschner M, Sigurdsson S, Anindya R, Reid J, Somesh B, Kong SE, Banks CAS, Conaway RC, Conaway JW, Svejstrup JQ 2009 Distinct ubiquitin ligases act sequentially for RNA polymerase II polyubiquitylation. Proc Natl Acad Sci U S A 106:20705–20710. doi:10.1073/pnas.0907052106. 19920177 PMC2778569

[54] Weems JC, Slaughter BD, Unruh JR, Hall SM, McLaird MB, Gilmore JM, Washburn MP, Florens L, Yasukawa T, Aso T, Conaway JW, Conaway RC 2015 Assembly of the Elongin A ubiquitin ligase is regulated by genotoxic and other stresses. J Biol Chem 290:15030–15041. doi:10.1074/jbc.M114.632794. 25878247 PMC4463447

[55] Yasukawa T, Kamura T, Kitajima S, Conaway RC, Conaway JW, Aso T 2008 Mammalian Elongin A complex mediates DNA-damage-induced ubiquitylation and degradation of Rpb1. EMBO J 27:3256–3266. doi:10.1038/emboj.2008.249. 19037258 PMC2609743

[56] Aso T, Lane WS, Conaway JW, Conaway RC 1995 Elongin (Siii): a multisubunit regulator of elongation by RNA polymerase II. Science 269:1439–1443. doi:10.1126/science.7660129. 7660129

[57] Clement JQ, Qian L, Kaplinsky N, Wilkinson MF 1999 The stability and fate of a spliced intron from vertebrate cells. RNA 5:206–220. doi:10.1017/s1355838299981190. 10024173 PMC1369753

[58] Grandvaux N, Servant MJ, tenOever B, Sen GC, Balachandran S, Barber GN, Lin RT, Hiscott J 2002 Transcriptional profiling of interferon regulatory factor 3 target genes: direct involvement in the regulation of interferon-stimulated genes. J Virol 76:5532–5539. doi:10.1128/jvi.76.11.5532-5539.2002. 11991981 PMC137057

[59] van Sluis M, McStay B 2017 Nucleolar reorganization in response to rDNA damage. Curr Opin Cell Biol 46:81–86. doi:10.1016/j.ceb.2017.03.004. 28431265

[60] Wallnerova Z, Albrecht P 1966 Reproduction of Tahyna virus in chick embryo cell cultures studied by biological and morphological methods. Acta Virol 10:140–148. 4380459

[61] Pichlmair A, Kandasamy K, Alvisi G, Mulhern O, Sacco R, Habjan M, Binder M, Stefanovic A, Eberle CA, Goncalves A, Burckstummer T, Muller AC, Fauster A, Holze C, Lindsten K, Goodbourn S, Kochs G, Weber F, Bartenschlager R, Bowie AG, Bennett KL, Colinge J, Superti-Furga G 2012 Viral immune modulators perturb the human molecular network by common and unique strategies. Nature 487:486–U101. doi:10.1038/nature11289. 22810585

[62] Kainulainen M, Habjan M, Hubel P, Busch L, Lau S, Colinge J, Superti-Furga G, Pichlmair A, Weber F 2014 Virulence factor NSs of rift valley fever virus recruits the F-box protein FBXO3 to degrade subunit p62 of general transcription factor TFIIH. J Virol 88:3464–3473. doi:10.1128/JVI.02914-13. 24403578 PMC3957945

[63] Andersen JS, Lam YW, Leung AKL, Ong SE, Lyon CE, Lamond AI, Mann M 2005 Nucleolar proteome dynamics. Nature 433:77–83. doi:10.1038/nature03207. 15635413

[64] Blakqori G, Weber F 2005 Efficient cDNA-based rescue of La Crosse bunyaviruses expressing or lacking the nonstructural protein NSs. J Virol 79:10420–10428. doi:10.1128/JVI.79.16.10420-10428.2005. 16051834 PMC1182624

[65] Habjan M, Penski N, Spiegel M, Weber F 2008 T7 RNA polymerase-dependent and -independent systems for cDNA-based rescue of Rift Valley fever virus. J Gen Virol 89:2157–2166. doi:10.1099/vir.0.2008/002097-0. 18753225

[66] Matrosovich M, Matrosovich T, Garten W, Klenk HD 2006 New low-viscosity overlay medium for viral plaque assays. Virol J 3:63. doi:10.1186/1743-422X-3-63. 16945126 PMC1564390

[67] Habjan M, Pichlmair A, Elliott RM, Overby AK, Glatter T, Gstaiger M, Superti-Furga G, Unger H, Weber F 2009 NSs protein of rift valley fever virus induces the specific degradation of the double-stranded RNA-dependent protein kinase. J Virol 83:4365–4375. doi:10.1128/JVI.02148-08. 19211744 PMC2668506

[68] Cheng C, Sharp PA 2003 RNA polymerase II accumulation in the promoter-proximal region of the dihydrofolate reductase and gamma-actin genes. Mol Cell Biol 23:1961–1967. doi:10.1128/mcb.23.6.1961-1967.2003. 12612070 PMC149466

[69] Livak KJ, Schmittgen TD 2001 Analysis of relative gene expression data using real-time quantitative PCR and the 2^–ΔΔ^*^CT^* method. Methods 25:402–408. doi:10.1006/meth.2001.1262. 11846609

